# Response Time to a Vibrotactile Stimulus Presented on the Foot at Rest and During Walking on Different Surfaces

**DOI:** 10.3390/s18072088

**Published:** 2018-06-29

**Authors:** Landry Delphin Chapwouo Tchakouté, Louis Tremblay, Bob-Antoine J. Menelas

**Affiliations:** 1 Department of Mathematics and Computer Sciences, University of Quebec at Chicoutimi, Chicoutimi, QC G7H 2B1, Canada; landry.chapwouo1@uqac.ca; 2 Department of Health Sciences, University of Quebec at Chicoutimi, Chicoutimi, QC G7H 2B1, Canada; louis1_tremblay@uqac.ca

**Keywords:** reaction time, response time, foot, vibrotactile stimuli, wearable device, enactive shoe, type of surface

## Abstract

This study investigates the simple reaction time (SRT) and response time (RT) to a vibrotactile stimulus presented on two body locations at the lower extremity of the foot on different types of surface during walking. We determined RTs while walking on Concrete, Foam, Sand, and gravel surface. Also, for RT, we evaluated two vibrotactile stimulus (VS) locations on the lower extremity: the ankle (AL) and under the foot plantar (FP). A total of 21 young adult participants (*n* = 21), aged mean 24 ± 2.9 years, took part in a two-session experiment with two main conditions (at rest and while walking on four types of surface). The control session included 2016 repeated measures, with one-way and two-way ANOVA analyses. The findings have consistently revealed slowness of RT to VS, in particular on sand and gravel surface. In addition, we found that body location has a significant effect on RT in certain surfaces. These results showed that RTs increased with environment changes during the performance of dual tasks.

## 1. Introduction

Aaron et al. defined RT as the time elapsed between the onset of a stimulus and the response to that stimulus [1]. Several types of RTs are defined related to their associated stimuli. For instance, auditory stimulus takes 8–10 ms to reach the brain [2] and mean auditory RTs are 140–160 ms [3,4,5]. In addition, visual stimuli take 20–40 m to reach the brain, with mean visual RTs being 180–200 ms [4,6]. Yet, RT to touch with the hand is intermediate at 155 ms [4,7]. Nevertheless, numerous factors, such as the environment, sensory motor diseases, fatigue, and age can influence RT and increase it, as reported by Kosinski [4], who presented a review on RTs noting that age is a factor affecting RT. In daily activities, RT plays an important role. For example, during walking, neural systems process different information, like vestibular, mechanoreceptor, visual, or auditory input, in order to continuously adapt walking patterns to the environment [1]. The real-time processing of this information is thus crucial for an adequate gait. As demonstrated by Mathis et al., visual information from at least two step lengths ahead is needed to guide foot placement when walking over complex terrain [8]. Other researchers have also considered the RT as a component of a model of prevention of falling [9]. Moreover, Lajoie and Gallagher [10] found that people who tended to fall in nursing homes had a significantly slower reaction time (RT) than those that did not. All these observations highlight the need to develop a means of preventing falls by evaluating RT. In fact, such observations have been the driving force for the study of RT in cases of balance disorders.

Neuropsychologists have named three basic kinds of RT experiments. In addition to simple reaction time (SRT), there are also recognition reaction time (RRT) and choice reaction time (CRT) [3,5]. As opposed to a SRT that implies a single stimulus and response, in RRT experiments, there are stimuli that should be responded to and certain distracting stimuli that should receive no response; the user has to react only to the right stimulus. In a CRT experiment, there are multiple stimuli, and each requires a different response. Apart from these three basics kinds of RTs, there may be a combination of SRT and CRT, as in the case of the probe reaction time (Probe RT) suggested by Posner and Boies [11]. The Probe RT requires subjects to perform a CRT task (primary task) with one hand and a SRT task with the other hand (secondary task) simultaneously. Based on the definition of RT provided in [4], we will situate our evaluation within the framework of a RRT. RT has been employed to collectively refer to all components of time required to complete a task after the appearance of a stimulus [12,13].

In this study, the term RT is rather inappropriate because we mainly measure the response time during a task where the central nervous system is already engaged. For the purpose of this study, we use the term RT to refer to response time during dual tasks (for instance, during walking and pressing a button on a smartphone), and the term SRT to RT in the rest position.

In the elderly, many falls occur on different types of surface [4] or when walking on a stairway [12]. We hypothesized that, possibly because of aging and the time to readjust the postural control, the information perceived by some mechanoreceptors among others could influence the RT of the person, as well as the types of surface on which they are walking. If falls are a major health problem, then a first study might be able to evaluate young people, and RT could be a solution to see how a healthy person responds to stimuli on different types of surfaces.

In fact, certain studies have improved the usability of RT in response to haptic stimuli. Cinaz et al. designed and evaluated a wearable device in order to assess RT to haptic stimulus [14]. Their study was performed with 20 subjects in idle conditions and under cognitive load. They measured the RTs of 10 subjects from a desktop-based RT test in the first half of the experiment. In the second half, they performed a wearable RT test. They observed that, in both experimental conditions, individual changes in RTs were augmented with cognitive load. Moreover, Ivorra et al. implemented haptic stimulus to investigate the central nervous system in a minimally obtrusive way [15]. In a first feasibility study, they showed that a SRT test can be continuously administered throughout the course of normal life activities. Recently, Peon and Prattichizzo studied the RTs to visual, auditory, and haptic stimulus while transmitting information [16]. They compared different sensory modalities (vibratory, auditory, and visual) and employed two intensities for the auditory and haptic modalities. For the visual modality, they used a screen in front of the users showing two colors—black and white—until the user retracted the tool. The results demonstrated that the haptic channel can provide faster RTs than the auditory one.

All these studies evaluated various aspects affecting RT, such as age, as presented in [4,17], or comparisons between different stimuli, as presented in [16]. Yet, a potential limitation for RT measure evaluation is related to the constraints of conveying VSs when walking on various flooring surfaces. In fact, the relationship between RTs and risk of falling is very complex. To the best of our knowledge, there is very little research concerning the RT to a VS when walking on various types of surface. Moreover, the feasibility of the technological measures of RT to VS are not elaborate enough, particularly when walking is divided by increasing the difficulty of the task of changing flooring surfaces. Therefore, it could be that some cueing, especially VS, could decrease or increase the risk of falling, especially on unstable surfaces. We addressed research questions utilizing a new, wearable enactive shoe capable of induction and measuring RT to VS on four types of surface. Next, the present study investigated the SRT and RT to a VS on two body locations at the lower extremity of the foot on varying types of surface during walking. Specifically, the purpose of this study is to assess factors affecting the RT with induced VS on the foot when walking on four types of surfaces. We also wanted to compare RTs to VS at two different positions.

## 2. Apparatus: Enactive Shoe

Recently, we have also designed an enactive shoe [18], controlled by a smartphone, that prevents falls related to a person’s immediate environment or an abnormal gait [19,20]. This device includes a set of sensors and actuators distributed in strategic positions. Some sensors are used to characterize the dynamics of walking [21,22] by measuring the speed and acceleration of the foot or the flexion of the shoe. Many of these sensors focus on measuring lateral and anteroposterior oscillations, as well as the distance between two strides, while other sensors provide information about the properties of the environment (humidity, temperature, inclination of the surface, dampness, and rigidity). This device is available, completely transparent to the user, inexpensive, non-invasive, and can be used to communicate a risk level of falling [23].

### 2.1. Characterization of The Haptuator

**Figure 1:**
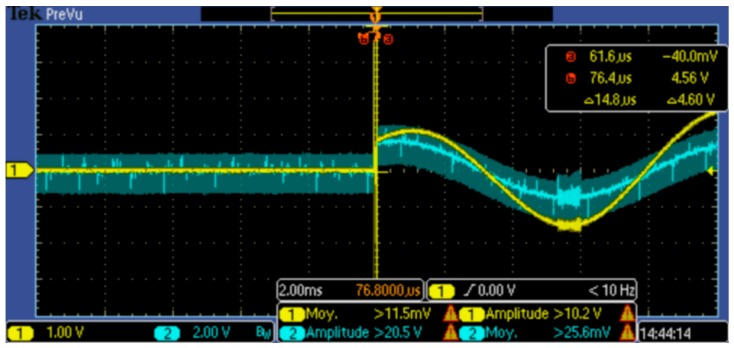
Characterisation test of the device displayed by an oscilloscope.

We characterized our device to evaluate the physical properties that could influence RT. Each engine produces an acceleration before reaching steady state. This latency can have a great influence on the perception of the stimulus, and therefore, on RT as well. We sought to determine the time that the engine takes before reaching its cut-off frequency and becoming stabilized. For this, we powered the motor (haptuator) by a function generator with an impedance of 10 ohms, providing a stable signal of 3.3 volts at a frequency of 100 Hz. The voltage (shown in yellow in Figure 1) is read by channel 1 of the oscilloscope. The current (in blue in Figure 1) is equivalent to the motor torque read on channel 2 of the oscilloscope and is programmed with amplification at an input of x100. The current is measured across a shunt resistance of 0.01 ohms in series with the motor. The display of the voltage of channel 2 therefore corresponds to 1 mV = 1 mA.

We can ignore the first impulse that is a “bounce” (a noise generated when the function generator is turned on). We can see that there is a delay (Figure 1) between the start of the signal (cursor a) and the initial rise of the motor current (corresponding to starting inertia). The current reaches its maximum at cursor b. This delay is approximately 19.6 μs. Further, this device-characterization test revealed that the Mark II haptuator has a latency of 0.0196 ms. However, such a delay is relatively minimal in terms of influencing RT, so that is why we decided later if we had to take it into account or not.

### 2.2. Positioning of The Haptuator On a Specific Body Location

Regardless of the limited research concerning the foot, it is known that the haptic modality is bidirectional, providing coupled and distributed returns across our body through the skin, muscle, and joint receptors [24]. To ensure this, we must make certain the sender transmits the message to the correct recipient on the appropriate channel using the code known by the sender and recipient. To ensure information communication, this model suggests identifying the components involved in the transmission of our VS, namely: communication device, the body location (point of contact of the stimulus with the skin), and the information to be transmitted. We know that VS can be transmitted through various body locations according to various devices [18], but it is important to specify the location of the stimulus. In fact, the sensory physiology of the FP is similar to that of the skin of the hand with the same types of tactile mechanoreceptors.

There are four main types of mechanoreceptors: slow-adapting type I (SA-I), slow-adapting type II (SA-II), fast-adapting type I (FA-I), and fast-adapting type II (FA-II) [24,25,26]. Various FA mechanoreceptors are located on lower extremities, for instance under the arch of the second toe and at the ankle. Our aim is to evaluate the RT to a VS using an enactive shoe embedding a haptic actuator (in this case, haptuator). It is a robust means to identify a specific body location of the stimulus such that the user must respond as quickly as they perceive the stimulus. For this, FA seemed to be suitable, as we needed fast transmission and rapid RT.

**Figure 2:**
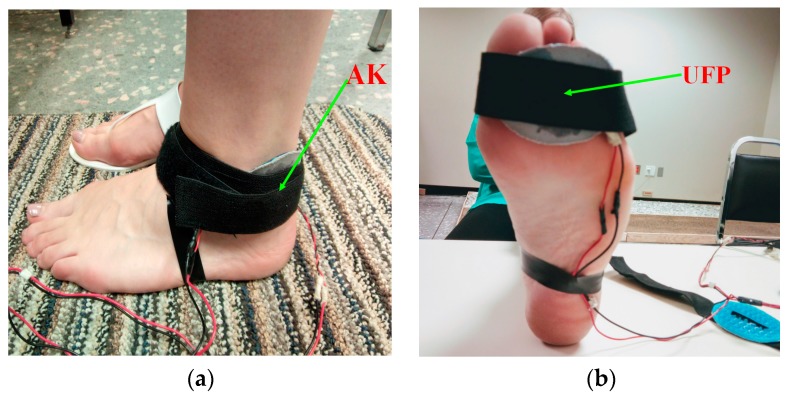
The device is worn on the left foot, and a strap holding the haptuator is located on the two lower extremities where the fast mechanoreceptors (FA-I and FA-II) are more represented in order to render VS. (**a**) In the first half of the experiment, the haptuator is located at the lateral ankle joint; (**b**) in the second half, haptuator is located under the arch of the second toe.

Previous studies have demonstrated that VS can be perceived through the foot. For instance, through an array of 16 dots of actuators, Velázquez et al. have shown that certain forms could be discriminated (stereognosia) in order to direct a blind person while walking [27]. Visell et al. investigated human differentiation and identification of haptic stimulus via the foot with vibration elements, like voice-coil actuators [28]. Recently, Velázquez et al. studied the communication of information and tactile-foot perception capability through human feet for assisting navigation [29]. The results show that subjects were capable of following directional instructions useful for navigating spaces. As such, the foot appears to be a suitable body location for haptic interaction and communication. Given this, FA-I were used to convey the stimulus as suggested in [27,30], and we selected two body locations where the fast mechanoreceptors (FA-I and FA-II) are more dense:

- The ankle (AL), especially between the lateral ankle joint (just behind both malleolus) and the Achilles tendon (Figure 2a)
- Under the FP, especially under the third toe of the foot (Figure 2b).

For the comfort of the user, the underside of the second toe of the foot is a hollow area, so the thickness of the actuator will have a less painful effect when walking.

### 2.3. Materials

In a previous study, we evaluated the impact of auditory distraction on the identification of VS presented under the FP [31]. We assessed whether having auditory disturbances would increase the learning time of vibrotactile messages. The VS was rendered throughout an enactive shoe with two haptuators. The findings highlighted the negative effect of auditory distractions on the number of iterations and completion time to recognize VS. The device was connected to a power outlet and participants were at the resting position. In addition, it was not possible to position the device at different places on the body. However, in walking, a wearable functionality device is necessary to ensure accuracy in conveying information using haptic modality when walking outdoors. Indeed, Yang et al. reported that wearables are mobile electronic devices that are worn on the body, or can be attached or embedded in clothes and accessories, such as Google Glass, or incorporated into the body, such as via smart tattoos, to supply a functional, portable and mostly hands-free electronic system [32].

**Figure 3:**
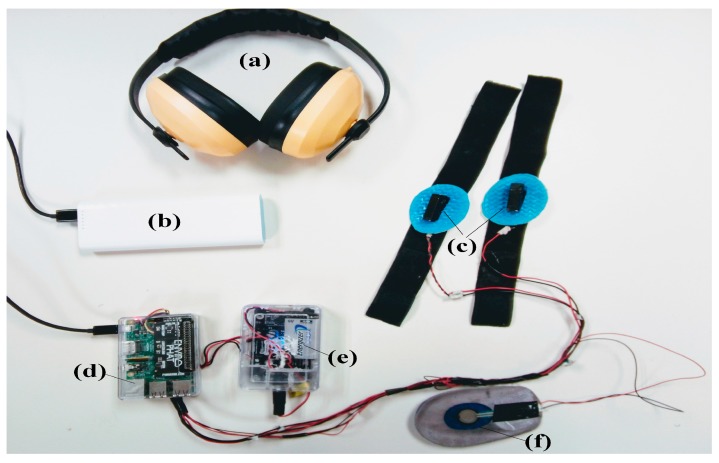
Enactive wearable and removable insole: (**a**) ear protection; (**b**) Power supply of 5 v; Haptic system ((**c**) Mark II haptuator mounted on strap; (**e**) Bluetooth amplifier with a battery); Computing system ((**d**) Raspberry Pi + EnviroPhat; (**f**) Force sensitive resistor).

For the purpose of this study, we designed a wearable device with a mini-computer embedded in its sensors and actuators that can display, process, or gather information, and have wireless communication capabilities. It can be attached to the body, communicate wirelessly with smartphones via the Bluetooth and Wi-Fi protocols, and convey VS when walking, to a specific location on the body. To this end, we employed certain components, mainly subdivided into two modules (Figure 3). Firstly, the design requirement was portability. As such, the device had to be attached or mounted onto the body during the experiment because participants were walking. Secondly, the VS had to be induced and perceived on a specific location on the foot while allowing movements of the foot. Thirdly, because of foot size variability, and as previously noted in Section 4.1, we designed a device with two separate enclosures, as suggested in [33]. Each enclosure is a module with a specific function (haptic and processing). In the first enclosure, the haptuators are mounted on removable straps that can be placed directly on a specific location. In the second, a computing unit is mounted. Each plastic enclosure of the device can be attached to the foot of the participant with a strap. The dimensions of each enclosure (Figure 3d,e) were: Width x Length x High (90 mm × 55 mm × 30 mm). The main function of each module is as follows:

#### 2.3.1. The Haptic System 

The haptic system (Figure 3c–e) is responsible for transmitting the VS to a specific location of the body. The system consists of an Android application on a smartphone and a Class D Bluetooth Amplifier powered by a 9 v lithium rechargeable battery (600 mAh). The input audio signal is transmitted by Bluetooth from the computing system. Then, it is amplified with a gain of 26 decibels before being sent to the two stereo outputs. At each stereo output, we connected a second-generation Mark II haptuator from TactileLabs [34]. Each haptuator measures 32 mm × 9 mm × 9 mm, with a frequency range between 90 to 1000 Hz, an impedance of 5.5 Ω, a maximum input voltage of 3.0 v, a maximum input current of 0.5 A, and a weight of 9.5 g. The maximum acceleration (7.5 G) is reached at 3 v. TactileLabs [34] reported that they provide an accurate signal and a faster time response (≈1 ms). The equipment is fixed onto the body of the participant and signal-driven wirelessly from the smartphone. The measurements were performed at 121 Hz, as the optimal response of the FA mechanoreceptor (Pacinian corpuscles) is noted to be at frequencies between 10–500 Hz [15].

#### 2.3.2. The Processing System 

The processing system (Figure 3d–f) is a built-in application with the main task of sending VS to the foot. The processing system consists of five main units:

- The Raspberry Pi 3 B (Figure 3d): The Raspberry Pi 3 Type B motherboard is a powerful and affordable solution for all types of compact or embedded systems. It is equipped with a Quad-Core ARM Cortex-A53 1.2 GHz processor (Broadcom (San Jose, CA, USA) BCM2837), 1024 MB RAM and a Dual-Core VideoCore IV GPU capable of decoding 1080 p HD video streams.
- Power supply (Figure 3b): we used a TP-LINK power bank model TL-PB15600 with dual-flexible output ports. It is powered with a 5 v rechargeable battery featuring a 15,600 mAh power bank.
- The EnviroPhat (Figure 3d): this is the analog unit used to recover the signals from the force-sensitive resistor, as well as the accelerations when the participants are walking.
- Force sensor (Figure 3f): Interlink 402 Force-Sensitive Resistor (FSR) with a detection range of 10 g up to 10,000 g for a resistance of 100 KΩ to 100 MΩ. There are two FSRs, positioned one each at the heel of the foot (rear FSR) and under the big toe (front FSR).
- A smartphone running the Android application which is used to record response time measurements when the participant presses on the tactile screen.

### 2.4. The VS Embedded in The Device

One VS of one second from a previous study evaluating SRT within the foot [10] is utilized. This stimulus was designed according to various studies of psychophysical perception reported in [21,31,32,35,36]. To meet the goal of the current work, we chose a pure sinusoidal wave with a waveform, as described by Equation (1); the signal is elicited at 121 Hz, as the optimal response of the vibration receptors (Pacinian corpuscles) is reported to be at frequencies between 10–500 Hz [25].

*W* = a sin(2π121t), (1)

## 3. Experiment

In this experiment, our objective was to investigate factors influencing RT to a VS while walking on four types of surface with the enactive device. During the first half of the experiment, the stimulus is sent to the AL (Figure 2a), and we evaluated RT. In the second half, the stimulus was sent to the FP (Figure 2b).

### 3.1. Participants

Twenty-eight students aged from 20 to 28 (24.2 ± 2.9 years) that were healthy, i.e., without musculoskeletal problems, from the University of Quebec at Chicoutimi (UQAC) participated in the study. They were recruited by means of accidental sampling after a written electronic invitation to participate in a study related to the RT and response time of a VS. All participants attended the session voluntarily and informed consent was obtained before experimental sessions. All assessments were performed in a controlled laboratory environment. Further, all participants were novices to haptic technologies. Each participant filled out a short questionnaire on their health history along with touch inspection surrounding their foot sensitivity. In the case of any foot sensitivity problems, the person was excluded from the study—seven participants were removed for this reason. Finally, only 21 participants without any sensorimotor deficits were retained because they presented tactile foot acuity and provided consent (Table 1). The experiment and consent form had was approved by the local ethics committee (certificate number: 602.434.01). To achieve the study goal, one experiment was carried out.

### 3.2. Experimental Setup

What follows is the description of the setup, including conditions.

#### 3.2.1. Test Environment

The experimental phase took place in a calm space, specifically, in our laboratory at UQAC, equipped with chairs and a table for preparation of the participants. The laboratory was equipped with the flooring surface conditions and a hygienic kit to clean the device after each session was finished. This environment remained constant during the experiments.

#### 3.2.2. Types of Surface

**Figure 4:**
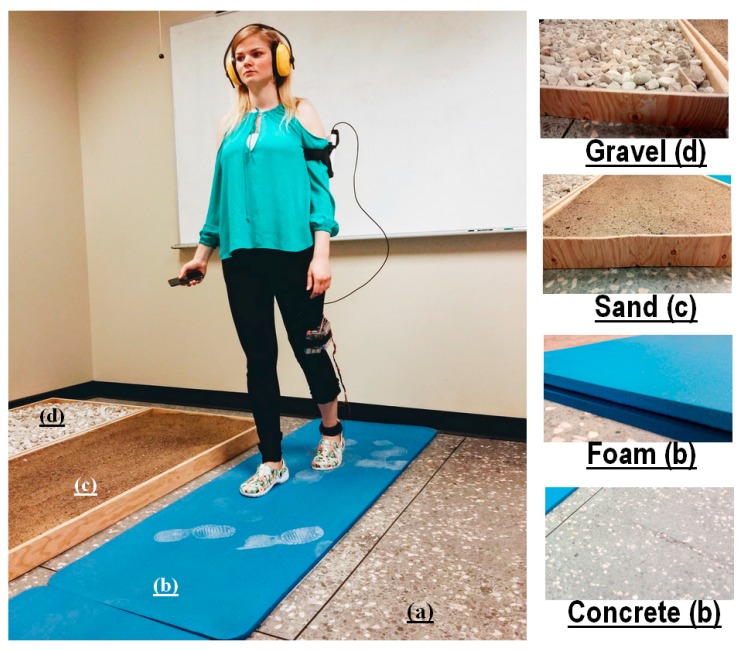
A participant performing the test wearing the enactive device and ear protection. The task consists of walking, focusing on the black spot on the opposite wall, and performing the RT test on the foam surface. Four types of surface named: concrete (**a**); foam (**b**); sand (**c**); and gravel (**d**).

The first factor (first condition) of the experiment was a set of several types of surface representing the natural flooring surface materials commonly found in daily life, such as concrete (Figure 4a), foam (Figure 4b), sand (Figure 4c) and gravel (Figure 4d). As shown in (Figure 4), we designed a longitudinal and wooden partitioning device to accommodate the gravel and sand surfaces (length = 350 cm; width = 71 cm; height = 7.5 cm). With this, foam surface is a little bit longer and less thick (length = 370 cm; width = 71 cm; height = 4.5 cm). Overall, all surface signified each of the five steps of the walking cycle. In particular, we filled each partition with real materials. The setup was the actual volumes of the corresponding materials (real gravel, real sand, etc.) placed in a longitudinal box.

### 3.3. Experimental Protocol

Our protocol is summarized in Table 2. Generally, we had two main sessions, that are described as follows:

#### 3.3.1. Experiment Sessions: Baseline and Control

To achieve the goal of this study, the experiments involved two sessions. The first concentrated on baseline treatment and the second on the control treatment. The baseline is the session where we collected SRT at a resting position on the concrete surface, whereas the control was the session where we collected RT on four types of surface during walking. Each session (baseline and control) featured familiarization followed up with a test phase. The first half of each test phase was concerned with collected measures when the stimulus location is at the AL (Figure 2a). During the second half, we changed the stimulus location toward the FP (Figure 2b). Within the entire session for each participant during each phase, we used two specific locations to apply the VS successively. When the stimulus was applied at the AL level, the RTs collected were named RT_AL. Under the arch of the second toe of the FP, the RTs collected were dubbed RT_FP. The order of stimulus location (AL and FP) was alternated for each participant between the sessions.

The control test for different types of surface was made up with 2016 measures (21 participants × 3 trials × 4 types of surface × 4 identifications × 2 positions × 1 vibrotactile stimulus). The overall length of a session was 45 min, with a break of 5 min between the two sessions.

#### 3.3.2. Familiarization Phase

**Figure 5:**
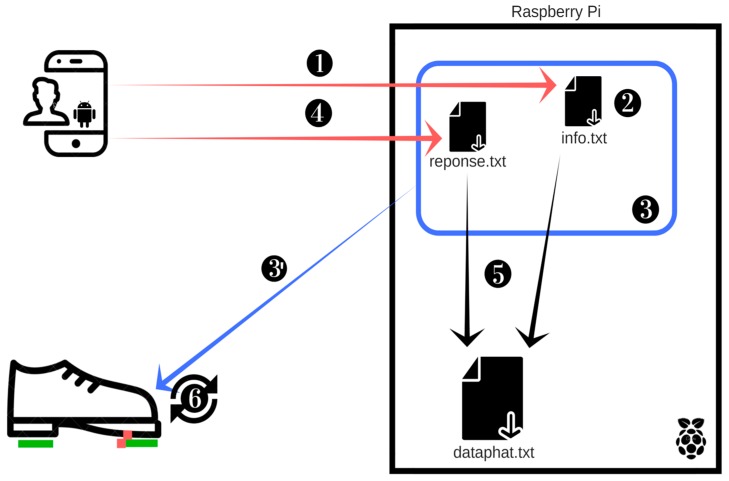
Experiment synopsis. 1. Sending the parameters of the experiment and information regarding the user in the processing system; 2. Saving settings and information in information.txt; 3. The processing system waits for the user to perform the first step; 3. The processing system detects the step and transmits FSR actuated to the haptic system, which sends the first VS (*t*_1_) and commences a delay of 5 s; 4. The user presses the screen and the time (*t*_2_) is recorded in the file reponse.txt; 5. The processing system retrieves the response, calculates the SRT and records it in dataphat.txt; 6. The processing system waits for the next step that will actuate the FSR.

The familiarization phase was concerned with the explanation and demonstration of the participants getting in touch with all aspects of the experiment. During this phase, the participants were at rest in a chair, wearing ear protection, and hosting the tactile device on the left foot. We chose the left foot for technical reasons. Nothing in the literature suggests that there may be differences in lower extremity perception due to dominance. To avoid false-positive perceptions on different types of surface, participants were encouraged to detect the VS in order to be familiar with them. A demonstration of the expected flow is located in Figure 5. Thereafter, we cleaned and reinstalled the system. The participants fixed on a black spot on the opposite wall in order that the screen in hand be outside of their field of vision. When they perceived VS on the foot, they pressed the smartphone screen as quickly as possible, and the time taken was stored. We recorded 25 measurements and generated a training chart of all RT measures. Once the participant was trained, they began with the testing phase.

#### 3.3.3. The Test Phase

The test phase that includes the baseline session was performed at rest, with the participants sitting on a chair, as shown in Table 2. However, the control session of the test phase was conducted while the participants were walking on the four types of surface (Figure 4). At this stage, participants donned ear protection. For each trial walk, at the initial time (*t*_1_), the processing system sends the VS four times. As soon as the participant perceived the VS, they pressed the smartphone screen. Thereafter, the identification time (*t*_2_) was saved and the processing system computed the *RT_i_* = *T_i_*_2_ − *T_i_*_1_ (*i* ≤ 4), where *i* represents the number of identifications per trial on one type of surface. When measures were completed on a given type of surface, the participant was invited to move on to the next. The trials on the different surfaces were counterbalanced (random) between participants. A synopsis of the trial with one type of surface is found in Figure 5.

## 4. Results and Discussion

Twenty-eight participants took part in this experiment to evaluate RT to VS, but seven were removed before the test because of documented sensitivity issues, limitations of the haptuator technology for haptic stimulation, or both. We used a wearable haptic device to convey the VS to two locations (FP and AL). All remaining participants went through the experiment successfully. On one hand, we observed the fastest RT_AL (98.79 ms) for participant N (male), and the fastest RT_FP (96.75 ms) for participant C (female) both on the concrete surface. On the other hand, the slowest RT_FP (600 ms) was observed for participant T (female) and the slowest RT_AL (595 ms) was observed for participant G (female), both on the gravel surface. Observations of the mean results are presented in Table 3; the fastest means were RT_FP (mean = 392.6; SD = 70.29) and RT_AL (mean = 465.43; SD = 80.02). Both these RTs were observed on gravel surface. Yet, the fastest means were RT_FP (mean = 146.62; SD = 32.21) and RT_AL (mean = 118.8; SD = 32.13). Both RTs were observed on concrete surface.

As we were investigating whether or not walking on a given surface type and/or the location of the haptuator had an impact the reaction time to a haptic stimulus, we may make the following assumptions:

- First (H_1_): Does the type of surface affect the RT?
- Second (H_2_): Does the position of the haptuator affect the RT?
- Third (H_3_): Is the SRT at the rest position for concrete surface different for FP and AL?

**Figure 6:**
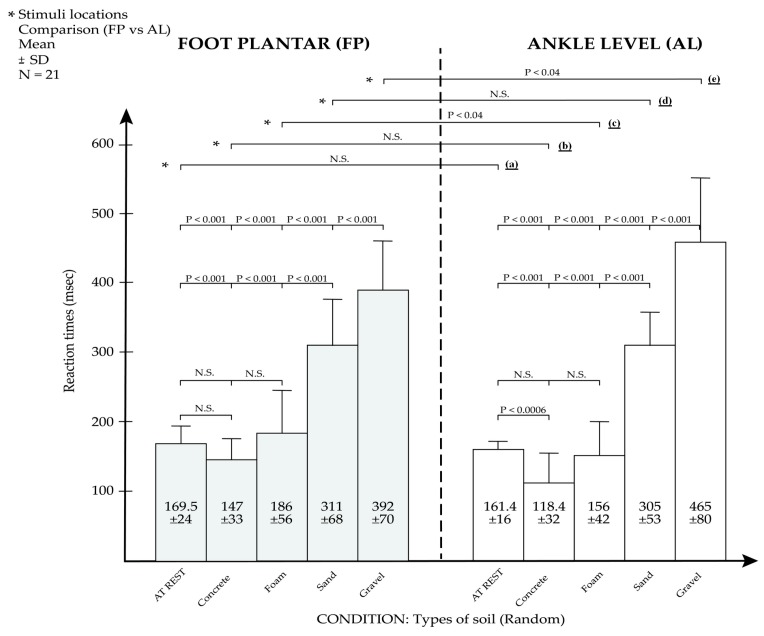
Comparison of reaction times on young participants at two location of stimulus (Foot plantar and Ankle). * are results from turkey comparison for the two-way ANOVA.

We assumed that for the null hypotheses H_01_, H_02_, and H_03_, all means were equal, and for the alternative hypothesis (H_a1_, H_a2_, and H_a3_), at least one mean was different from another. Our significance level is (alpha) = 0.05. The dependent variable is RT, and our independent variables are types of surfaces and stimulus location. We have more than two groups, and the variables are quantitative. All tests were performed using GraphPad Prism version 5.02 for Window (GraphPad PRISM, San-Diego, CA, USA). All analysis of variance (ANOVA) evaluations were performed with post-hoc Tukey HSD (Honest Significant Difference) tests. Our input data were *k* = 4 independent first group factors, concrete, foam, sand, and gravel. The second group factors were FP and AL. The sample observation was *n* = 21. The data satisfied the conditions to justify the use of ANOVA. All Tukey comparisons are illustrated in Figure 6.

Figure 6 show that there is a strong trend for SRT at rest being slower (about 20%) than response time when subjects are engaged in a complex motor task such as walking. Figure 6 also shows that response time is considerably slower when subjects are walking on sand (by about 225%) and gravel (by about 311.6%). The VS location site either at the foot plantar (FP) or at the ankle (AL) only effect by 20% the RT to VS at AL site versus RT to VS at FP site.

### 4.1. Effect of Types of Surface on RT(H_1_): Do the Type of Surface Affect the RT?

To evaluate hypothesis H_1_, One-way ANOVA was conducted for the two conditions (FP and AL) with repeated measures. Results from the ANOVA-FP test showed that the *p*-value corresponding to the F-statistic of the one-way ANOVA was lower than the α level (F(3,80) = 77.46, F_critical_ = 2.72, *p* < 0.0005, Effect sizes ($\eta_{\mathrm{ufp}}^{2}$) = 0.75), suggesting that one or more surfaces were significantly different when the stimulus was presented at the FP. Moreover, results from the ANOVA-AL test showed that the *p*-value corresponding to the F-statistic of the one-way ANOVA was lower than the α level (F(3,80) = 174,45, F_critical_ = 2.72, *p* < 0.0005, Effect sizes ($\eta_{\mathrm{ufp}}^{2}$) = 0.75), suggesting that one or more types of surface were significantly different. These results are also reported in Figure 6, revealing that the type of surface had a significant effect on RT. RTs were faster on concrete and foam surfaces, but slower on sand and gravel. This indicates that certain surfaces have more of an influence on RTs than others. However, one could consider the influence of double tasks (walking and pressing a button with one’s hand) to explain the slowed RT on sand and gravel surfaces (Figure 6). That said, we believe that a number of variables, like visual, vestibular, and auditory sensory systems, and others such as verbal instruction, were not controlled for this study and can explain the difference in RTs on various types of surface. Indeed, during the experiment, we noted that participants tended to look at the surface for a few seconds when they were walking, and sometimes slightly lost balance owing to the uneven flooring surface and gait disturbance. This indicates that the RT would have been influenced by a visual and external stimulus [31,35]. Moreover, this is why we suggested the implication of neurocognition (variables such as a difference in knee muscle extension strength and proprioception, haptic touch, balance, and interlimb coordination) that elucidates the differences observed between experimental conditions, in particular by divided attention and the possibility of risk of falling. All this suggests that RTs are influenced by the ability of subjects to pay attention (divided attention) and concentrate on the dual task, but also to increase the difficulty of the task (walking on various types of surface) with a potential increase in risk of falls in young participants.

### 4.2. Stimulus Location and Types of Surface (H_2_): Do the Position of the Haptuator Affect the RT?

To evaluate the hypothesis, H_2_, two-way ANOVA was conducted for the two conditions—the stimuli location (FP and AL) and types of surface (concrete, foam, sand and gravel) with repeated measurements. Furthermore, we assessed the positioning of the haptuator when walking on four types of surfaces. For the two-way ANOVA results, there was no significant difference for location of the stimulus, F(1,160) = 0.07, *p* = 0.797. Yet, there was a statistically significant difference between various types of surface factor means, (F(3,160) = 236.99, *p* < 0.0005). In addition, there was a significant interaction between the effects of location and types of surface on RT, F(3,160) = 7.52, *p* < 0.0005. The results (Table 4 and Table 5, Figure 6 suggest that stimulus location (FP or AL) had no influence on RT (*p* > 0.05) when participants walked on concrete or sand (Figure 6, (b) and (d)). This is based on the fact that on sand, participants tended to have almost the same mean RT (FP mean RT = 311 ± 68 ms; AL mean RT = 305 ± 53 ms). However, interactions between the locations and other surfaces (foam and gravel) revealed significant results (Figure 6, (c) and (e)). As such, this demonstrates a weak significant effect for the interaction between stimuli location and type of surface. However, this is not really surprising. We suggest that the complexity of the walking task on sand can reduce tactile perception. For concrete and stimulus location, the explanation could be that concrete is a form of hard-flooring surface where one has almost the same average speed when walking. In addition, the level of difficulty is almost nil, because people are used to walking on that type of flooring surface every day. Moreover, for both locations, the covered distance of the VS (conduction time) was about ±17–20 cm between the two stimuli locations. Hence, if the density of haptic receptors were similar on both sides and the nerve conduction velocity (sensory and motor) was 100 m per second, this variation in RT would be relative, between 15–20 ms, because of the difference in distance between stimulus location sites. This variation represents a very small effect which is lost in a global variability of RT. Yet, all participants have almost the same RT mean and standard deviation on sand owing to the balance on this type of soft surface that has the same level of difficulty for all participants. Indeed, the shorter RTs found here confirmed that the FA mechanoreceptor of the foot (FP and AL) (Figure 2) are robust locations for stimulation to evaluate RT.

### 4.3. SRT at Rest (H_3_): Is the SRT at the Rest Position on Concrete Surface Different on FP and AL?

To evaluate hypothesis H_3_, a one-way ANOVA was conducted for the two conditions (FP and AL) with repeated measures. The statistical results according to Figure 6. Comparison of reaction times on young participants at two location of stimulus (Foot plantar and Ankle). * are results from turkey comparison for the two-way ANOVA. Figure 6 (a) performed to analyze SRT at rest were not significant (*p*-value > alpha-level). The SRT were collected on a concrete surface at rest. This means that the SRT did not change while in a static position, even if we modified the location of the haptuator.

To the researchers’ knowledge, this is the first study to examine SRT to VS stimulation on the lower limb extremities (short vibration at the foot). In the past, one of us [36] measured the SRT at rest in 10 young adults without musculoskeletal problems following a short burst of 100 Hz electrical stimulation (ES) of 50 m at a two-time threshold of sensory detection. The ES was made on the skin in the forefoot in level five (L5) dermatomes. The voluntary response of dorsal foot flexion was recorded using electromyography (EMG) activity in extensor digitorum brevis muscle. However, in the present work, the SRT for FP was 169 ± 24 ms and for AL was 161.4 ± 16 ms. This suggests that measurements with new technologies are valid. However, we cannot propose a different haptic density of the haptic receptors between FP and AL (concrete = 147 ± 33 vs. 118 ± 32) in RT conditions to compare (169.5 ± 24. vs. 161.4 ± 16) in SRT conditions to explain the significant difference in concrete surface in terms of AL and FP stimulation.

### 4.4. Comparison of SRT and RT

Results from the ANOVA-FP testing showed that the p-value corresponding to the F-statistic of the one-way ANOVA was lower than at the α level [F(3,80) = 77.46, F_critical_ = 2.72, *p* < 0.0005, Effect sizes ($\eta_{\mathrm{ufp}}^{2}$) = 0.75], suggesting that one or more surfaces were significantly different when stimulus is presented at the FP. In addition, results from ANOVA-AL testing indicated that the *p*-value corresponding to the F-statistic of the one-way ANOVA was lower than the α level (F(3,80) = 174,45, F_critical_ = 2.72, *p* < 0.0005, Effect sizes ($\eta_{\mathrm{ufp}}^{2}$) = 0.75), suggesting that one or more types of surfaces were significantly different. These results can be explained as follows.

Response time for the concrete surface during a dual task (walking and pressing a smartphone) when we consider both sites of VS was on average 20.2% (147 ± 33 ms vs. 118.4 ± 32 ms) shorter than SRT at rest. This is different than in the literature, where complex RTs were consistently longer than SRT [4,36]. However, in our methodology, the physical environment and instructions were controlled. We believe that during walking, the neurocognitive attentional process was under dynamic engagement of the central nervous system (frontal lobe), facilitating sensory-motor cortex (afferent and efferent information systems), brain stem, and spinal cord response (already in action) for movements (walking).

Walking is a complex motor act, a multi-segmental task and a semi-automatic control of rhythmic movements by the central nervous system. Indeed, it is under cortical control, but also of the cerebellum, pedunculopontine nuclei in the upper brainstem, and local interneuron networks in the spinal cord. This implies that sensory information (haptic and other) coming from lower limbs reach in real time and continuously, during walking, the sensory cortex, SI and SII, and then the motor cortex, MI and MII, and almost simultaneously, the prefrontal cortex (cognitive and attentional process), to correctly analyze and execute the directive of pressing the smartphone screen quickly after haptic feeling on the foot, and, thereafter, motor cortical and the sub-cortical efferent response reach motor spinal cord pathways. This latency represents the response time. During motor action (walking), this neurological network is facilitated during vibrotactile stimulation, and explains why RT is shorter than SRT, or, in other words, during walking, the time for motor preparation and movement initiation were facilitated. Moreover, in a preliminary study [35], we investigated six young adult subjects with a similar protocol, except that subjects feeling VS were asked to lift one foot (dorsal flexion) as quickly as possible. The experiment was interesting because this task represented the motor preparation and motor initiation of movement during motor conflict during the gait cycle. The subjects had to assure safety balance and avoid the risk of falling before lifting the foot. Our results demonstrated that the response time for lifting the foot quickly and pressing the smartphone after VS perception during walking were: on concrete surface—147 ± 33 ms and 118.4 ± 32 ms; on foam—186 ± 56 and 156 ± 42 ms; on sand—311 ± 68 ms and 305 ± 53 ms; and on gravel—392 ± 70 ms and 465 ± 80 ms. Nevertheless, in the prior study, where participants had to lift one foot quickly after perception of VS, we observed great variability of the mean RT and standard deviation compared to the present study, because a lengthy duration was necessary to analyze walking safely with respect to balance in a timely fashion. We suggest that the long time for analyzing is to avoid or reduce the risk of falls.

The protocol involved with lifting the foot quickly after the VS [35] represented principally a walking perturbation compared to the present study. During a perturbation process, we used other neurologic strategies to resolve the situation. As such, we corrected the motor task by including a hand task as the dual task. In our opinion, this preliminary study suggests that RT is not a stereotyped response, and that environmental information is more important than haptic information for control of movement, such as walking.

Overall, the significant results showed that the RT varied according to the type of surface, with a major propensity for gravel, while there was a minor propensity observed for concrete. We also found that another factor influencing the RT to VS is the stimulus location. These two factors (type of surface and stimulus location) can be added to the list of factors influencing the RT found in the literature [4]. However, it was found that hand RT reduced relative risk of falls using a visual stimulus and a finger-press response by 21% [37] and 31% [38], respectively. Nevertheless, in the present study, the VS was presented at the foot level and the RT mean was not higher than for other studies [37,38]. Therefore, as our mean RT values were fast on certain types of surfaces, we suggested that evaluation of RT to VS on the foot can be interpreted as a physiological characteristic of humans to diminish fall risk. Finally, we know that RT slows with age; RT involves both motor and cognitive processing, and a short RT is required for responding to environmental changes or perturbations of the center of mass [38].

## 5. Conclusions and Future Works

The main objective of this study was to investigate, for the first time with new technologies, the simple RT (at rest) and the response time to a short haptic stimulus on the lower limb while walking. The results revealed the feasibility of using a new device (enactive shoe and smartphone) capable of conveying VS and recording response time at two locations of the lower limb (AL and FP). The findings suggested that RT is faster on concrete and foam when compared to sand and gravel surfaces. The SRTs at rest were 20% slower than the response time with regards to the control condition (concrete surface) during dual-task conditions (walking and pressing smartphone). The stimulus location has a weak effect, especially on the gravel. One possible extension of this work would be to use an apparatus more adapted to convey VS, for instance, adding more Mark II haptuators at different locations under the FP. More people organized in two groups (fallers and non-fallers), the elderly, or some motor disease patients with impairment of balance like, Parkinson’s disease (PD) patients will be recruited for a future study to increase the significance of results with respect to effect of age and sex. Finally, another possible extension of this study would be to recruit the elderly or some motor disease patients with impairment of balance, like PD patients, and study the impact of age or impairment within two groups (faller and non-fallers).

## Figures and Tables

**Table 1:** Participant’s characteristics

Participants	Value
Age (Y)	24.42 ± 2.87 *
Height (cm)	168.47 ± 10.64 *
Weight (kg)	68.24 ± 11.66 *
Gender	Men (*n* = 8)|Women (*n* = 13)

* Values are represented as Mean ± Standard deviation (SD).

**Table 2:** Experiment protocol summary.

Sessions	Conditions				
	At Rest	Walking			
	C	C	F	S	G
Baseline	Familiarization phase *				
	Test phase				
Control		Familiarization phase *			
		Test phase	Test phase	Test phase	Test phase

* Familiarization was made only on the Concrete surface for all sessions. C = Concrete, F = Foam, S = Sand, and G = Gravel surface.

**Table 3:** Means and standard deviations of reaction times, showing results when reaction time is obtained at rest position and when participants were walking.

		At Rest	Walking			
		Concrete	Concrete	Foam	Sand	Gravel
FP ^1^	Mean	169.5	146.6	186.2	311.3	392.6
	SD ^3^	24	33.2	56,3	68.5	70.2
AL ^2^	Mean	161.4	118.8	156.6	305	465.4
	SD ^3^	15.9	3.1	42.2	53.7	80

^1^ FP = Reaction time (RT) obtained when stimulus is presented at the Foot Plantar. ^2^ AL = RT obtained when stimulus is presented at the Ankle. ^3^ SD = Standard deviation.

**Table 4:** Two-way ANOVA results.

Source	DF	Seq SS	Contribution	Adj SS	Adj MS	F-Value	*p*-Value
Locations	1	215	0.01%	215	215	0.07	0.797
Types of surface	3	2,307,063	79.56%	2,307,063	769,021	236.99	0.000
Locations * Types of surface	3	73,210	2.52%	73,210	24,403	7.52	0.000
Error	160	519,190	17.91%	519,190	3245		
Total	167	2,899,677	100.00%				

**Table 5:** Two-way ANOVA coefficient: distance between factor levels and the overall mean.

Term	Coef	SE Coef	95% CI	T-Value	*p*-Value	VIF
Constant	260.36	4.39	(251.68; 269.04)	59.24	0.000	
Locations						
AL	1.13	4.39	(−7.55; 9.81)	0.26	0.797	1.00
FP	−1.13	4.39	(−9.81; 7.55)	−0.26	0.797	*
Types of surface						
Concrete	−127.65	7.61	(−142.68; −112.61)	−16.77	0.000	1.50
Foam	−88.88	7.61	(−103.91; −73.84)	−11.68	0.000	1.50
Gravel	168.66	7.61	(153.63; 183.69)	22.16	0.000	1.50
Sand	47.86	7.61	(32.83; 62.89)	6.29	0.000	*
Locations * Types of surface						
AL Concrete	−15.04	7.61	(−30.07; −0.01)	−1.98	0.050	1.50
AL Foam	−15.94	7.61	(−30.97; −0.90)	−2.09	0.038	1.50
AL Gravel	35.28	7.61	(20.24; 50.31)	4.63	0.000	1.50
AL Sand	−4.30	7.61	(−19.33; 10.73)	−0.56	0.573	*
FP Concrete	15.04	7.61	(0.01; 30.07)	1.98	0.050	*
FP Foam	15.94	7.61	(0.90; 30.97)	2.09	0.038	*
FP Gravel	−35.28	7.61	(−50.31; −20.24)	−4.63	0.000	*
FP Sand	4.30	7.61	(−10.73; 19.33)	0.56	0.573	*

* Variance inflation factor (VIF) are not available for that row.
